# Depression among people with dyspepsia and *H*. *pylori* infection: A community based cross-sectional study in Ethiopia

**DOI:** 10.1371/journal.pone.0275424

**Published:** 2022-10-06

**Authors:** Matiwos Soboka, Esayas Kebede Gudina, Mulatu Gashaw, Hiwot Amare, Melkamu Berhane, Hailemichale Desalegn, Dagimawi Tewolde, Mulusew Gerbababa Jebena, Solomon Ali, Andreas Wieser, Guenter Froeschl, Markos Tesfaye

**Affiliations:** 1 Department of Psychiatry, Medical Faculty, Jimma University, Jimma, Ethiopia; 2 Department of Internal Medicine, Jimma University, Jimma, Ethiopia; 3 School of Medical Laboratory Science, Jimma University, Jimma, Ethiopia; 4 Department of Pediatrics and Child Health, Jimma University, Jimma, Ethiopia; 5 Department of Internal Medicine, St. Paul’s Hospital Millennium Medical College, Addis Ababa, Ethiopia; 6 Faculty of Public Health, Department of Epidemiology, College of Health Sciences, Jimma University, Jimma, Ethiopia; 7 Department of Microbiology, Immunology and Parasitology, St. Paul’s Hospital Millennium Medical College, Addis Ababa, Ethiopia; 8 Division of Infectious Diseases and Tropical Medicine, University Hospital, Ludwig-Maximilians-Universität, Munich, Germany; 9 German Center for Infection Research (DZIF), Partner Site Munich, Munich, Germany; 10 Department of Psychiatry, St. Paul’s Hospital Millennium Medical College, Addis Ababa, Ethiopia; Duke University Medical Center: Duke University Hospital, UNITED STATES

## Abstract

**Background:**

Depression is the most common mental health problem, and frequently associated with physical illnesses. A link between depression, dyspepsia and Helicobacter pylori (*H*. *pylori*) infection has previously been reported. However, there is limited data regarding the association between these conditions from sub-Saharan Africa where they are highly prevalent.

**Objective:**

This study aimed at elucidating the potential associations between depression, dyspepsia and *H*. *pylori* infection in Ethiopia.

**Methods:**

We conducted a community based cross-sectional study involving urban and rural residents aged 13 years or older in Jimma Zone, southwest Ethiopia. A total of 871 participants were evaluated using a structured case reporting format for symptoms of dyspepsia and the patient health questionnaire (PHQ-9) for depression. Additionally, participants were assessed for *H*. *pylori* infection using stool antigen and serology tests. A multivariate logistic regression was used to identify the association between depression, dyspepsia and H. pylori infection after controlling for potential confounders.

**Results:**

The prevalence of PHQ-9 scores indicative of probable case of depression among all participants was 10.9%. The prevalence of probable case of depression among patients who had at least one symptom of dyspepsia was 13.3% (X^2^ = 15.1 = p-value<0.001), while it was 11.9% (X^2^ = 1.23, p-value = 0.26) among patients who had *H*. *pylori* infection. Out of patients who took medications for their heartburn in the past 30 days, 14.9% (X^2^ = 3.6, p-value = 0.06) had probable case of depression. Dyspepsia symptoms such as epigastric discomfort (aOR = 2.59, 95%CI = 1.14, 5.87), postprandial fullness (aOR = 1.70, 95%CI = 1.48, 5.51), nausea (aOR = 1.71, 95%CI = 1.04, 2.82) excessive belching (aOR = 0.53, 95%CI = 0.31, 0.92) were associated with probable case of depression. However, being *H*. *pylori* test positive, gender, and age were not associated with probable case of depression.

**Conclusions:**

There was an increased prevalence of probable case of depression among patients who had dyspepsia symptoms and *H*. *pylori* infection. Longitudinal studies are needed to examine possible further determinants of association between symptoms of dyspepsia and probable case of depression.

## Introduction

Depression is mainly characterized by feelings of sadness and lack of interest with other supporting symptoms over a longer period of time [1]. Depression is the most common mental health problem affecting one in five people over their lifetime [2]. Estimates on point prevalence suggest that about 300 million people suffer from depression globally [3]. Depression was ranked as the third major cause of global burden of disease in 2008 [3] and second leading cause of Disability Adjusted Life Years in 2020 [4]. Depression is predicted to become the globally most important burden of disease by 2030. Also, depression has a detrimental impact on all aspects of sustainable development goals [5]. Among patients with mental health problems, depression is the primary root cause of suicide and somatic diseases [3]. Moreover, somatization is frequently encountered in depressive disorders [6]. Depression frequently affects individuals with various physical health conditions such as cardiovascular, gastrointestinal or infectious diseases [2–5].

*H*. *pylori* infection is a common cause of upper gastrointestinal symptoms and affects people of all ages. It is more prevalent in developing countries as the risk of acquiring this infection is mostly associated with lower living standards and socioeconomic status [7]. It is estimated that about 50% of people are affected worldwide. The transmission route of *H*. *pylori* is not fully understood but fecal/oral or oral/oral exposures are identified modes of transmission [8].

There are several possible biological pathways linking depressive symptoms, *H*. *pylori* infection and GI symptoms including endocrine, nervous, and immunological system. First, at times of stress the Hypothalamus-Pituitary-Adrenal (HPA) axis becomes activated in response to the stress as an adaptation mechanism. However, chronic stress can result in a maladaptive state of elevated levels of cortisol potentially contributing to mood disturbance [9–11] as well as to the development of upper GI symptoms. Second, there is a recognized interaction between the gut microbiota and the CNS functions [12]. Studies have reported that the change in the gut microbiome could cause emotional disturbances, behavioural changes [13] as well as mental health problems including depression, but the mechanism is still under investigation [14]. Third, depression may potentially influence the gut through changes in the functions of autonomic nervous system and activating the HPA axis including corticotrophin-releasing factor and cortisol [15–18]. In addition, psycho-bio-immunological pathways played a role, where patients suffering from depression show impaired immune function, and this could lead to an increased disposition to infectious diseases, including infections with H. pylori [19].

*H*. *pylori* infection has a strong association with psychiatric disorders particularly anxiety and depression [20]. A systematic review done by *Al Quraan*, *A*. *M*. *et al* reported an association between depression and *H*. *pylori* infection [8]. Depression is common among patients with gastritis [21] and associated with gastrointestinal inflammation [22] as well as functional dyspepsia [23]. Depression is also one of the risk factors for functional dyspepsia, gastric adenoma/carcinoma, and irritable bowel syndrome [15]. Similarly, patients with dyspepsia were found to have a three-fold increased risk of developing major depression [24]. Furthermore, individuals with adjustment disorder may experience physical symptoms as a reaction to the stressful events, and this may overlap with the symptoms of dyspepsia. The imbalance between life stressors and coping skills may mediate the gastric disturbances which manifest as symptoms of functional dyspepsia [25]. People with adjustment disorder may also report symptoms of depression in addition to the physical symptoms [26].

Although depression commonly coexists with *H*. *pylori* infection and dyspepsia, patients do not receive treatment early. Depressed patients often present primarily with physical complaints, and the underlying psychiatric morbidity is often overlooked by healthcare workers. This leads to under-diagnosis, delay in the treatment of depression, and unnecessary treatment for presumed causes of the somatic symptoms. Investigating the association between depression, dyspepsia, and *H*. *pylori* infection may provide a better understanding for better intervention strategies to improve the treatment outcomes of patients with dyspepsia or *H*. *pylori* infection. Therefore, this study aimed at elucidating the association between depression, dyspepsia, and *H*. *pylori* infection in a community setting of a low-income country.

## Methods

### Study design and settings

This is a cross-sectional study conducted on a sample of adults and children drawn from urban, semi-urban and rural communities in Southwest Ethiopia.

### Selection of study participants

This study is a part of a study conducted to assess the sero-epidemiology of *H*. *pylori* infection, dyspepsia, and their associated factors in Southwest Ethiopia. Individuals aged 13 years or older were recruited. Participants were classified into three age categories: 13–19, 20–29, and ≥30 years. The number of participants was determined based on the age-group stratified risk of acquiring *H*. *pylori* infection. The sample size was calculated for each of these categories using Epi Info^™^ Version 7.2.4.0 using the sample size formula for population survey. Assumptions taken into account to calculate sample size were population size of >10,000 for each category; 5% margin of error; and expected proportion of *H*. *pylori* infection of 10% for school-age (3–6.5 years) [27], and 52% for the remaining age groups [28]. This yielded a total of 1422 participants (both children and adults) for the original study.

### Sampling procedure

A multi-stage and cluster sampling technique was used to select participants from randomly selected nine *woreda*s (districts) in Jimma Zone. A total of 28 *kebele*s (the lowest administrative unit in Ethiopia), of which 24 from rural *kebele*s and four from an urban setting, were then randomly selected. Finally, households (HH) with adolescents and adults were randomly selected in each *kebele*. The first HH was randomly selected from the lists of HHs registered by health extension workers (HEW) in each *kebele*. Once the first HH was selected, the next HH at a walking distance was approached and assessed for eligibility. If there was no eligible participant from the HH, the next house replaces it. This was continued until the final sample size allocated for that *kebele* was achieved. Due to intra-cluster correlation within a household, we used parallel sampling techniques for adults and adolescents. If the selected HH had more than one eligible person, a lottery method was used to select one study participant per family. Only apparently and self-declared healthy individuals who gave consent and were willing to provide both blood and stool samples were recruited. Individuals who consented to participate in the study but were unable to give blood and/or stool specimens at the time of data collection were excluded from the final analysis.

### Data collection procedures

#### Assessment for dyspepsia

Participants were interviewed for symptoms of dyspepsia and related gastrointestinal (GI) conditions. Dyspepsia was defined as the participant reporting occurrence of one or more of the following symptoms persisting for at least the past three months–epigastric pain, epigastric discomfort, epigastric burning, postprandial fullness, early satiation, abdominal bloating, heartburn, acid regurgitation, and excessive belching.

#### *H*. *pylori* antibody test

A 3–5 ml blood sample was collected for *H*. *pylori* serology and stored in the refrigerator at 2–8°C (for a maximum of 6–8 hours) until the transport to the Jimma University Medical Center laboratory, where *H*. *pylori* antibody tests were performed. The blood samples were centrifuged at 1500 rpm for 15 minutes, and the plasma was separated from which *H*. *pylori* antibody test was done. The antibody test was performed using Core tests^®^ ONE STEP TEST KIT (Core Technology Co., Ltd., Beijing, China), a rapid *H*. *pylori* antibody test kit for the qualitative detection of IgG antibodies specific to *H*. *pylori* in serum or plasma.

#### Stool antigen test

About 0.5 grams stool specimen was collected in a dry container to test for the presence of *H*. *pylori* antigen. All the stool samples were evaluated at the site of collection for the presence of *H*. *pylori* antigen using Wondfo^®^ One Step *H*. *pylori* Antigen Feces Tests Cassette (Guangzhou Wondfo^®^ Biotech Co., Ltd., Guangzhou, China) according to the manufacturer’s instructions.

For the scope of this study, a confirmed *H*. *pylori* infection was defined as a positive result either in a specific serum antibody test and/or a stool antigen test.

#### Probable case of depression

The patient health questionnaire (PHQ-9) was used to assess probable case of depression. The tool entirely depends on patient self-report. This tool was validated in Ethiopia to assess probable case of depression in different settings [29–31]. PHQ-9 has 9 items with a total possible score of 27. Score 1–4 indicate minimal depression; score 5–9 indicate mild depression; score 10–14 indicate moderate depression; score 15–19 indicate moderately severe depression; score 20–27 indicate severe depression. The mean score and variance of PHQ-9 were 1.4 and 6.01 respectively. In this study a score of 5 or more was used to define probable case of depression [32].

### Data management and analysis

The collected data was cleaned and entered into EpiData Entry version 3.1. The data was then imported to SPSS^®^ Statistics version 21 (IBM^®^, New York, USA) for analysis. Descriptive statistics were used to present patient characteristics. First, we performed frequency of depression across socio-demographic characteristics. Also, we computed Chi-square test to see if there is a significant difference between the expected and observed frequencies. Bivariate analysis was done to assess the association between single independent variables and the outcome variable (probable case of depression). Marital status, education, family size, and occupation were not included in the final model due to overly missing values. All variables with a P-value less than 0.20 in the bivariate logistic regression were entered together into a multivariable logistic regression in order to control for potential confounders such as age, gender, and other socio-demographic variables. Variables with P-values of 0.05 or lower were considered to be associated with the outcome variable. The final model was adjusted for socio-demographic variables, and previous history of treatment for dyspepsia to see the association between probable case of depression and dyspepsia symptoms and H. pylori infection. The strength of association of the variables was presented as odds ratio with its 95% confidence interval.

### Ethical considerations

The study was approved by the Institutional Review Board of Jimma University Institute of Health (IHRPGD/3012/18). Written informed consent was obtained from adults (≥18 years of age). For younger participants (13–17 years of age), both the consent of the guardian and assent from the participants was obtained before participation in the study.

## Results

### Socio-demographic and clinical characteristics

A total of 871 participants were included in this study, 57.9% of which were females. The mean age of the study participants was 29.9 years (SD = 12.8) with a range of 13 to 80 years. The majority of the study participants were married (61.7%; n = 537), did not have any formal education (67.2%; n = 585), and were rural residents (84.6%; n = 737).

Overall, 74.1% (n = 645) of the participants had at least one symptom of dyspepsia, while 57.9% (n = 504) were positive for *H*. *pylori* by either serology or stool antigen test (Table 1).

**Table 1:** Socio-demographic characteristics and probable case of depression among patients with dyspepsia in Ethiopia (n = 871).

Variables	Frequency N (%)		Probable case of depression		p-value
			No N (%)	Yes N (%)	
Gender	Male	367(42.1)	332(90.5)	35(9.5)	0.27
	Female	504(57.9)	444(88.1)	60(11.9)	
Marital status	Single	251(28.8)	236(94.0)	15(6.0)	0.05
	Married	537(61.7)	468(87.2)	69(12.8)	
	Divorced	34(3.9)	29(85.3)	5(14.7)	
	Widowed	45(5.2)	39(86.7)	6(13.3)	
Education	No formal education	585(67.2)	532(90.9)	53(9.1)	0.12
	Primary	120(13.8)	101(84.2)	19(15.8)	
	Secondary	100(11.5)	88(88.0)	12(12.0)	
	Tertiary	60(6.9)	50(83.3)	10(16.7)	
Residence	Urban	134(15.4)	122(91.0)	12(9.0)	0.43
	Rural	737(84.6)	654(88.7)	83(11.3)	
Occupation	Civil servant	63(7.2)	52(82.5)	11(17.5)	0.03
	Student	171(19.6)	164(95.9)	7(4.1)	
	Farmer	381(43.7)	332(87.1)	49(12.9)	
	Unemployed	77(8.8)	69(89.6)	8(10.4)	
	Others	144(16.5)	128(88.9)	16(11.1)	

### Probable case of depression and associated factors

The prevalence of probable case of depression among the study participants was 10.9% (n = 95). The prevalence of scores that would correspond to minimal, mild, moderate, and moderately severe probable case of depression among the total participants were 25.8% (n = 225), 9.5% (n = 83), 1.3% (n = 11), and 0.1% (n = 1) respectively.

Among the patients who had at least one symptom of dyspepsia, 13.3% (n = 86) had scores indicative of probable case of depression. In more detail, mild, moderate, and severe probable case of depression was observed in 11.6% (n = 75), 1.6% (n = 10), and 0.2% (n = 1) of patients who had at least one symptom of dyspepsia, respectively. Out of patients who were *H*. *pylori* positive (serum and/or stool test), 11.9% (n = 60) had probable case of depression.

Out of patients who took medications for their dyspeptic symptoms within the 30 days prior to the interview, 14.9% (n = 26) had probable case of depression. Even though the prevalence of probable case of depression was higher among females as compared to males, the difference was not significant (11.9% vs 9.5%, p = 0.27). Furthermore, about one in seven divorced patients had probable case of depression (14.7%; 5/34). The prevalence of probable case of depression among civil servant participants was 17.5% (n = 11) (Table 1).

On bivariate analysis, upper abdominal symptoms (COR (Crude Odds ratio) = 2.49, 95%CI = 1.57–3.96), epigastric pain (COR = 2.47, 95%CI = 1.72, 4.29), epigastric discomfort (COR = 3.63, 95%CI = 2.10, 6.41), and feeling full after eating small (COR = 3.37, 95%CI = 2.14, 5.29) were associated with probable case of depression (Table 2).

**Table 2:** Factors associated with probable case of depression among patients with dyspepsia in Ethiopia.

Variables		Multivariable analysis			
		aOR	P-value	95% CI	
				Lower	Upper
Gender	Male				
	Female	1.09	0.73	0. 68	1.72
Residence	Urban				
	Rural	-		-	-
Upper abdominal symptom	No				
	Yes	1.01	0.99	0.43	2.33
Epigastria pain	No				
	Yes	1.48	0.37	0.63	3.48
Epigastric discomfort	No				
	Yes	**2.59**	**0.02**	**1.14**	**5.87**
Epigastric burning	No				
	Yes	0.67	0.29	0.31	1.42
postprandial fullness	No				
	Yes	**1.70**	**0.002**	**1.48**	**5.51**
Feeling full after eating small	No				
	Yes	0.74	0.37	0.38	1.43
Heart burn	No				
	Yes	0.95	0.86	0.54	1.68
Acid regurgitation	No				
	Yes	-		-	-
Bloating	No				
	Yes	1.26	0.42	0.73	2.18
Excessive belching	No				
	Yes	**0.53**	**0.02**	**0.31**	**0.92**
Nausea	No				
	Yes	**1.71**	**0.03**	**1.04**	**2.82**
Ever taking medication for dyspepsia	No				
	Yes	1.43	0.18	0.85	2.39
H-pylori test result (serology and/or stool antigen test)	Negative				
	Positive	1.19	0.46	0.75	1.88

In the final model, epigastric discomfort (aOR = 2.59, 95%CI = 1.14, 5.87), postprandial fullness (aOR = 1.70, 95%CI = 1.48, 5.51), and feeling of nausea (aOR = 1.71, 95%CI = 1.04, 2.82) were associated with probable case of depression. Excessive belching was associated with lower odds of being a probable case of depression (aOR = 0.53, 95%CI = 0.31, 0.92). Age, gender, other symptoms of dyspepsia, previous treatment for dyspepsia, and being positive for a *H*. *pylori* test were not associated with probable case of depression (Table 2).

## Discussion

This study is the first to investigate the association between dyspepsia and probable case of depression in Ethiopia. In this study, symptoms of dyspepsia such as epigastric discomfort, postprandial fullness, and nausea were associated with probable case of depression. But, excessive belching was inversely associated with probable case of depression. The prevalence of probable case of depression among the participants overall was 10.9%, while it was 11.9% among *H*. *pylori* seropositive patients. There was also a higher prevalence of probable case of depression among those who were taking medications in the past. However, being positive in at least one of the two used tests (serology and/or antigen) for *H*. *pylori* infection was not associated with probable case of depression.

During our literature search, we could only identify a single study regarding depression among dyspepsia patients conducted on the African continent, a study in Kwa-Zulu, which used Depression, Anxiety and Stress Scale—21 Items (DASS-21) to assess depression. The prevalence of probable case of depression among patients with dyspepsia (13.3%) found in our study was much lower than in the study conducted in KwaZulu-Natal province (47.76%) [33]. Similarly, a study conducted in Malaysia as well reported a higher prevalence (22.6%) of depression among patients suffering from dyspepsia, using the Hospital Anxiety and Depression Scale (HADS) [34]. The difference between the three studies may be due to differences in the tools that were used to assess depression (PHQ9 vs DASS-21 and HADS). The tools used in the studies conducted in KwaZulu-Natal and Malaysia measure anxiety in addition to depression. Also, we have used a cross sectional study design, while the study conducted in Malaysia employed a case control design. Furthermore, the studies conducted in KwaZulu-Natal and Malaysia selected participants among individuals presenting to healthcare facilities who tend to have greater psychiatric morbidity than community settings. The prevalence of depression among patients with treatment refractory (63.3%) and non-refractory (20.9%) functional dyspepsia found in the systematic review done by *Esterita et al*. was also higher than our finding [35]. However, it is noteworthy that in a similar study on Chinese participants conducted in Hong Kong (12.4%) and mainland China (15.8%) the revealed prevalence were comparable to what we determined now in Ethiopia [24, 36].

In this study, there is an association between dyspepsia symptoms and probable case of depression which is in line with a systematic review done by *Al Quraan AM* et al that showed dyspepsia patients have higher rate of depression [8]. Likewise, the finding of this study is in line with a study conducted in Hong Kong that found dyspepsia to be associated with depression [24]. In more detail, in our study, the odds of showing signs of depression among patients with epigastric discomfort was three times higher than that of patients free of epigastric discomfort which is in line with studies conducted in Hong Kong, and in Yirga Cheffe in Ethiopia, that found patients with dyspepsia were three times at higher risk of depression [24, 37]. This is supported by the results of a prospective cohort study conducted in Australia which found patients with functional dyspepsia who reported minor depression symptoms at baseline developed severe depression after one year follow-up (mean difference = 1.08) [16].

The prevalence of probable case of depression found among patients with *H*. *pylori* infection (11.9%) in this study was lower than the findings of studies conducted in Turkey (24.1%) [38] and the Kingdom of Bahrain (32.1%) [20]. The difference may again be related to the tools used to assess depression (PHQ-9 vs Composite International Diagnostic Interview (CIDI) and Hamilton Depression Rating Scale (HDRS)). CIDI is in line with the clinical diagnostic criteria of Diagnostic and Statistical Manual of Mental Disorders (DSM-V) than PHQ-9 which is a screening tool for depression. CIDI’s validity is limited to clinical samples, but a clinical sample overestimates the prevalence of depression compared to a community sample [39]. Also, PHQ-9 is simple and reliable in measuring the severity of depression and can be used as an alternative to HDRS [40, 41]. HDRS has been commonly used to screen depression for a long time but some of its items have limitations in identifying the severity of depression [42]. Furthermore, our study sample was selected from a general community population, while the study done in Turkey and Kingdom of Bahrain selected participants from health facilities. As a consequence, there may have been a selection bias among patients at health facilities, for example due to a presumably higher prevalence of comorbidities which also involves mental health conditions when comparing to patients selected from the community. Likewise, depression is common among patients with other physical health problems [43].

In summary, there have been numerous studies worldwide that were able to indicate associations between dyspepsia and *H*. *pylori* infections on the one side and mental health impairment on the other side. However, a socio-cultural or anthropological in-depth investigation of potentially modifying factors that are rooted in the respective local cultures and settings is missing. We as an interdisciplinary study group of mental health and infectious disease specialists in Ethiopia will from this point work towards a further elucidation of this interconnection between gut and brain and the possible role of the socio-cultural setting.

To our knowledge, this is the first study in Ethiopia to assess the association between probable case of depression, dyspepsia, and *H*. *pylori* infection by including a large number of participants. Data were collected from different sites in Southwest Ethiopia so that the findings of this study could be generalized for all patients with dyspepsia and *H*. *pylori* infection in Southwest Ethiopia except admitted patients. Since a cross-sectional study design was used in this study, it is difficult to assess the temporal or causal relationship between dyspepsia symptoms, probable case of depression, and *H*. *pylori* infections. Further studies will be needed, both for elaborating on causal pathways between psychiatric disorders such as depression and dyspepsia, and for identifying potentially important social or patient characteristics as effect modifiers in an Ethiopian setting. The tool we used to assess depression in the study is a screening tool and not a diagnostic tool so that we anticipate the limitations in the accurate diagnosis of depression. The study participants may not report symptoms of probable case of depression due to fear of stigma which we did not address in this study. Also, the associations may have been due to confounding from nutritional deficiencies and other medical illnesses that are known to have relationship with feature of depression. However, we believe these are unlikely because of the apparent health status of the participants. With regard to the establishment of diagnosis of *H*. *pylori* infection, the serological assays that we have employed do not differentiate well between past and current infection and may therefore have led to an overestimation of *H*. *pylori* prevalence in our cohort. Moreover, although both the test kits used to diagnose *H*. *pylori* infection have been approved for use in Ethiopia by the Ethiopian Food and Drug Authority, published research articles or indications by the manufacturers on sensitivity and specificity for both tests were not available. However, the use of such test assays without proper validation procedures corresponds to the reality on the ground in our setting. The findings of this study may not be generalized to settings outside of our study setting, which was a community based study in the Southwest region of Ethiopia.

## Conclusions

We have shown positive associations between a range of symptoms indicative of dyspepsia and probable case of depression. Epigastric discomfort, Nausea, and postprandial fullness were associated with probable case of depression. For *H*. *pylori* infection, as established by the laboratory assays used, we could show only a trend, which was, however, also pointing towards more frequent positive probable case of depression scores in patients with *H*. *pylori* infection. Patients with symptoms of dyspepsia and/or *H*. *pylori* infection need be screened for probable case of depression using a quick and sensitive screening tool such as PHQ-9. However, the diagnosis of depression should be left to health care workers trained in mental health.

## Supporting information

S1 File (SAV)
